# Editorial: Single-molecule studies of DNA–protein interactions collection 2021

**DOI:** 10.1093/nar/gkab497

**Published:** 2021-06-19

**Authors:** Piero R Bianco, Julian E Sale, Rodrigo Reyes-Lamothe

**Affiliations:** Department of Pharmaceutical Sciences, College of Pharmacy, University of Nebraska Medical Center, Omaha, NE 68198-6025, USA; Division of Protein & Nucleic Acid Chemistry, MRC Laboratory of Molecular Biology, Francis Crick Avenue, Cambridge CB2 0QH, UK; Department of Biology, McGill University, 3649 Sir William Osler, Montreal, QC H3G 0B1, Canada

*NAR* is pleased to present a collection of 12 Original, Methods, and Survey and Summary articles highlighting the use of single-molecule microscopy in the study of DNA–protein interactions. The inspiration for this collection came from the Fusion conference, ‘DNA and Interacting Proteins as Single Molecules – *In Vitro* and *In Vivo*’, held in February 2020 in the Bahamas, attended by 47 participants from around the world. The collection brings together papers from that meeting with other recent work published in the Journal from this exciting field. The development and rapid improvement of single-molecule approaches are providing a new perspective to the understanding of DNA–protein enzyme kinetics, giving mechanistic insights that simply cannot be generated by more conventional bulk-phase assays. The present collection explores the diversity of currently available methods and discusses their potential for the discovery of novel molecular mechanisms.

Since their inception, single-molecule methods have evolved rapidly from the ability to study between 1 and 50 individual molecules at a time, to the study of hundreds, if not thousands in a single assay. These approaches, initially developed for the analysis of proteins and DNA *in vitro*, have also been extended to the study of individual proteins in single living cells. Coupled with the advances in single-molecule techniques are rapid advances in microfluidics from devices with single fluid streams to multi-stream and more complex designs with buffer wells. Further, while many groups utilize one single molecule approach, multiple groups often combine approaches into one instrument to increase our understanding of many cellular processes, for example magnetic tweezers combined with single-molecule fluorescence or, Fluorescence Resonance Energy Transfer (FRET) and optical tweezers.

To enable the reader to better understand how single molecules are manipulated, the review by Bianco and Lu (1) summarizes five widely-used approaches in their most simple formats before delving into the mechanistic insights that have been gained into how stalled DNA replication forks are rescued. The review highlights for readers references in which existing methods have been modified or that combine distinct single-molecule techniques. Similarly, Spakman *et al.* (2) summarize how parallel approaches have been used to understand the activity of Type 1A topoisomerases.

One of the earliest single-molecule microscopy methods for the study of DNA and the mechanisms of its processing enzymes is magnetic tweezers, by which one end of a DNA molecule is attached to a coverslip surface while the other is coupled to a super-paramagnetic bead trapped by a magnetic field. Changes in the position of the bead report on the activity of proteins on DNA. Öz *et al.* (3) used a technique derived from magnetic tweezers, called molecular DNA forceps, where the ends of two DNA molecules are maintained in close proximity by a third DNA molecule that serves as a bridge. Using DNA forceps, they characterized the activity Ku and LigD from the bacteria *Bacillus subtilis* during non-homologous end-joining.

Another set of techniques, including optical tweezers, use surface attachment to hold one end of DNA stationary then stretching DNA fibres in the field of view while tracking the interaction of single copies of proteins by fluorescence microscopy. Xue *et al.* (4) used a version of this approach called DNA curtains. Here, multiple molecules of DNA, stretched parallel to each other, are visualized to study the activity of the human helicase RECQ5 on single-stranded DNA. Spinks *et al.* (5) studied the kinetics of the bacterial replicative helicase DnaB at the replication fork by attaching one end of DNA to the surface of the coverslip and using a rolling circle DNA replication assay. In the two works mentioned above, DNA is attached to the surface of a coverslip and stretched by fluid flow, with imaging facilitated by wide-field and total internal reflectance microscopy, respectively. Barnet *et al.* (6) use an alternative approach where DNA molecules are immobilized and stretched between 5μm beads stuck to a surface (tightrope assay), to study the TFIIH subunits p44/p62 involved in nucleotide excision repair.

FRET is the basis for yet another set of fluorescence-based techniques. The proximity of fluorophores attached to DNA or proteins reports on the kinetics of binding and conformational changes occurring during the processes studied. Mazumder *et al.* (7) use single-molecule FRET (smFRET) and a site-specific, RNA polymerase labelling method using unnatural amino acids, to characterize the dynamics of the RNA polymerase clamp, a structural element implicated in the initiation of transcription. Mustafa *et al.* (8) and Sujay *et al.* (9) combined smFRET and site-specific labelling of DNA substrates to test length-dependent compaction of telomeres and the dynamics of the *E. coli* resolvase RuvC during Holliday junction resolution, respectively. In addition to the changes in intensity reporting proximity, smFRET experiments provide a wealth of kinetic information spanning multiple imaging channels.

Initially, the data sets from smFRET studies were small. As these have grown in size and the level of understanding increased, improved methods for extracting as much information as possible are required. To achieve this, Tibbs *et al.* (10) developed an analysis package called the kinetic event resolving algorithm (KERA). This algorithm organizes tracks from a range of multi-color, single-molecule experiments into groups based on transition patterns and displays dwell-time data. This enables the researcher to search for patterns of transitions between states and by analysing these in greater detail, ascertain whether they are artifacts of chance or the result of a biochemical transition.

Single-molecule fluorescence microscopy is being increasingly applied to the study of the kinetics of protein-DNA interactions in live cells. These methods rely on the substantial difference in the diffusion coefficients of proteins in ‘solution’ and DNA-bound proteins—which behave similarly to chromosomal loci. Brown *et al.* (11) summarize the use of *in vivo* single-molecule microscopy to study the function of Polycomb group proteins in mammalian cells. While huge progress has been made in multiple areas, the distinction between proteins in ‘solution’ and DNA-bound proteins remains challenging. Analysis methods often work by assigning thresholds to traits, such as speed and intensity, in fluorescent foci to identify DNA-bound proteins. However, these methods do not account for inherent fluctuations in the data and can lead to subjective choices and inconsistencies in the analysis, especially when molecules are tracked for an extended period of time. To address this issue, Kapadia *et al.* (12) developed Bound2Learn, a machine learning-based approach that uses the output from widely used tracking software packages, to enable the robust classification of tracks. Fully automated and accurate determination of residence times from live imaging will enable further discoveries of protein activities in cells.

It is evident that *in vitro* and *in vivo* single-molecule approaches have provided insight into the biochemical mechanism at a previously unattainable level of resolution. Much work remains as researchers find novel and exciting ways to combine single-molecule approaches, to further advance their techniques and improve analysis methods. When combined with the similarly advancing fields of software development for data analysis, microfluidics, and super-resolution microscopy, which is progressing towards real-time imaging at ever-increasing resolution, this exciting field promises to reveal only greater levels of insight into complex, multistep biochemical pathways of DNA processing.
